# Supplementary material for: Collapsing Glomerulonephritis in a Kidney Transplant Recipient after mRNA SARS-CoV-2 Vaccination
Source: J Clin Med. 2022 Jun 24;11(13):3651. doi: 10.3390/jcm11133651 (PMC9267596; doi:10.3390/jcm11133651)
Supplement: Supplementary file 1 [file jcm-11-03651-s001.zip › jcm-1772557-supplementary.pdf]

Supplementary Materials

Collection and management of Serum and Peripheral Blood Mononuclear Cell (PBMC) samples

PBMCs were isolated from patient's peripheral blood by Ficoll density gradient centrifugation and subsequently frozen in liquid nitrogen until their use in functional analyses. Serum was isolated by centrifugation and samples were stored at -20ºC.

Assessment of Spike specific IgG-producing memory B cells

To differentiate circulating memory B cells (mBCs) to antibody-secreting cells (ASCs), peripheral blood mononuclear cells were cultured (1.5×10^6 cells per ml at 37 °C in 5% CO2) for 6 days in Iscove Modified Dulbecco Media (IMDM) enriched medium, 500 ng/ml Human CD40/TNFRSF5 Antibody (Bio-Techne R&D Systems, S.L.U., USA), 600 IU/ml human interleukin-2 (Sigma Aldrich, USA), 100 ng/ml human interleukin-21 (Peprotech, UK), 25 ng/ml human interleukin-10 (Peprotech, UK), 2.5 ug/ml CpG-B DNA (ODN 2006) (HycultBiotech, The Netherlands) and 10 ul/ml ITS Liquid Media Supplement (Sigma Aldrich, USA), as previously described [1].

After 6-day stimulation, 4.5×10^5 stimulated cells were seeded in each well to assess SARS-CoV-2 specific IgG spots, whereas 4.5×10^4 and 4.5×10^3 stimulated cells were seeded to assess the polyclonal IgG spot detection.

For the detection of specific SARS-CoV-2 mBCs, we used RBD-WASP at 1:20 dilution; followed by anti-WASP-HRP at 1:1000 dilution. For the polyclonal IgG detection, mAbs MT78/145-Biotin (1 ug/ml) and Streptavidin-HRP (1:1000) were used, respectively.

Next, 100 uL of ready-to-use TMB (3,3′ , 5,5′-tetramethylbenzidine) solution was used as substrate for HRP, in order to develop the reaction until distinct spots emerge. After the plate was dried, spots were count in the Fluorspot Reader version 8 (AID® Gmbh, Strassberg, Germany). The ratio between RBD-specific mBCs over the total polyclonal IgG mBCs in each patient is used as a reliable approach to characterize the proportion or enhancement of a given RBD-specific IgG-antibody secreting cell (ASC) within the global IgG-ASC population.

Any ELISPOT test with non-detectable RBD-specific spots is considered as negative when assessed in a qualitative manner.

Assessment of Spike Glycoprotein specific T cell responses

Circulating Spike-reactive cytokine-producing memory T-cell frequencies were evaluated using a multicolor FluoroSpot Immune assay (AID® Gmbh, Strassberg, Germany), in which 3 distinct cytokine-producing T-cell frequencies were simultaneously assessed: effector (IFN-γ), proliferative (IL-2), central (IFN-γ/IL-2). Briefly, $2x10^5$ PBMCs (in 100 μl) were stimulated with the peptides for 24 hours. After washing steps, the different cytokine fluorospots were detected using primary and secondary antibodies against each cytokine plus the addition of enhancer. The spots obtained were automatically counted with the Fluorospot Reader version 8 (AID® Gmbh, Strassberg, Germany). The results were considered after subtracting to each well the responses obtained in the respective negative control wells. In each test, complete medium alone (20% Fetal Bovine Serum (FBS) and 80% RPMI solution) and Pokeweed (PWM) mitogen were used as negative and positive controls, respectively. We

previously showed [2], that unexposed individual to SARS-CoV-2 did not respond to any of the used overlapping peptide pools of SARS-CoV-2 proteins. Any antigen-specific ELISPOT test with less than 5 spots/2x10^5 PBMC was considered as negative when assessed in a qualitative manner.

These responses were evaluated against the Spike Glycoprotein (S) overlapping peptide pool (P0DTC2 protein, S gene), which contains 158 + 157 peptides of >70% purity and was designed by and purchased from JPT Innovative Peptide Solutions (JPT®, Berlin, Germany). This peptides were reconstituted in DMSO and PBS and used at a final concentration of 2μg/mL.

Supplementary References

1- Luque S, Lúcia M, Crespo E, Jarque M, et al. A multicolour HLA-specific B-cell FluoroSpot assay to functionally track circulating HLA-specific memory B cells. *J Immunol Methods*. 2018;462(July):23-33. doi:10.1016/j.jim.2018.07.011

2- Favà A, Donadeu L, Sabé N, et al. SARS-CoV-2-specific serological and functional T cell immune responses during acute and early COVID-19 convalescence in solid organ transplant patients. *Am J Transplant*. 2021. doi:10.1111/ajt.16570
